# Using music as a mood regulator in everyday life is associated with unfavourable health and fitness outcomes in overweight adults

**DOI:** 10.1371/journal.pone.0317607

**Published:** 2025-02-27

**Authors:** Laura Ginström, Kaisa Kaseva, Juha E. Peltonen, Suvi Saarikallio, Mari Tervaniemi

**Affiliations:** 1 Centre of Excellence in Music, Mind, Body, and Brain, Faculty of Educational Sciences, University of Helsinki, Helsinki, Finland; 2 Sports and Exercise Medicine, Faculty of Medicine, University of Helsinki, Helsinki, Finland; 3 Helsinki Sports and Exercise Medicine Clinic (HULA), Foundation for Sports and Exercise Medicine, Helsinki, Finland; 4 Department of Education, Faculty of Educational Sciences, University of Helsinki, Helsinki, Finland; 5 Centre of Excellence in Music, Mind, Body, and Brain, University of Jyväskylä, Helsinki, Finland; 6 Cognitive Brain Research Unit, Department of Psychology, Faculty of Medicine, University of Helsinki, Helsinki, Finland; Virginia Tech: Virginia Polytechnic Institute and State University, UNITED STATES OF AMERICA

## Abstract

Individual traits and habits have shown to be associated with health and health behaviour. One such habit is how individuals use music. This study aimed to examine if using music as a mood regulator is related to risk factors of lifestyle diseases. Participants who joined the present Motivation Makes the Move! lifestyle intervention were overweight and sedentary adults (n = 76, ages 19–40). They answered questionnaires about physical activity and use of music. They also underwent a cardiopulmonary exercise test and their body composition was assessed. Additionally, the analyses’ robustness was tested through controlling for physical, sociodemographic and psychological health related factors. We observed that despite the participants’ self-reported commitment to regular physical activity, their fitness level was poor. Using music as a mood regulator was associated with lower cardiorespiratory fitness. Use of music was also positively linked to body fat percentage, although this finding did not remain significant after adjusting for age, educational level and experienced health. We urge future research to address the relationship between music use and risk factors of lifestyle diseases in a population sample.

## Introduction

Overweight and obesity are risk factors for many lifestyle diseases, such as diabetes [1], cancer [2], musculoskeletal diseases [3], and cardiovascular diseases [4]. Physical inactivity and poor cardiorespiratory fitness are also connected to metabolic syndrome [5–7], which refers to a set of physiological risk factors (abdominal obesity, high blood pressure, high blood sugar, high triglyceride levels) that frequently occur together and are clearly connected to type 2 diabetes, cardiovascular disease, and higher mortality [8,9]. In addition, there are associations between obesity, psychiatric conditions, and decreased quality of life [10].

Physical inactivity is linked to overweight and obesity, and increasing physical activity can prevent and treat obesity [11]. While physical inactivity on its own is connected to poor physical fitness, multiple lifestyle diseases, and higher mortality, cardiorespiratory fitness is a better and an independent predictor of cardiovascular disease [12,13]. Cardiorespiratory fitness can be described by maximal oxygen uptake (VO_2max_), which indicates the body’s ability to transport oxygen from the breathing air to the working muscles and the capacity of muscle tissue to use oxygen. Efforts to improve cardiorespiratory fitness, encourage physical activity, and reduce overweight and obesity can take place at a societal or at a more personal level (e.g., through examining the motivations, needs, and behavioural choices of individuals).

It is known that individual traits influence health and health behaviour (see, e.g., [14–16]). Our interest in an individual’s lifestyle is in the use of music in everyday life. The health and well-being benefits of music, both listening and engaging in music making, have been shown in multiple studies [17]. Music seems to affect the cardiovascular system directly, having beneficial effects on for example heart rate variability (for a review, see [18]). Recently, there has also been evidence of the positive effect of music on exercise [19–21]. Music can help when preparing for sports and exercise [22], to cope during the process [23], and to recover better afterwards [24]. Actively engaging in music making can also help people with chronic pain to exercise by easing anxiety [25] and can help older adults exercise for a longer time [26]. It is plausible that an individual’s relationship to music may be connected to exercise and physical activity and possibly to the risk factors of lifestyle diseases.

In addition to its potentially positive effects on exercise, music can be useful and used in emotion regulation. For instance, listening to music has been rated as the second best means of emotion regulation after exercise out of 18 different activities [27]. Music also has stress-reducing effects [28], which may be relevant in the prevention of lifestyle diseases, as stress is linked to both overweight and metabolic syndrome [29]. The strategies of music-based emotional self-regulation range from distraction to mental processing [30]. Music listening and the related emotional self-regulation can help in coping with anxiety and can increase life satisfaction, e.g., [31,32]. Music listening can improve mood and support coping, particularly when the listening experience contains distraction from worries, cognitive reframing of experiences in a positive manner, or both [33–35]. Emotion regulation goals are typically best achieved in the daily moments when help is most needed, namely when the episode of music listening begins with the listener experiencing a negative affective state [36].

In this study, we were interested in exploring the relationship between everyday use of music in mood-regulation and the risk factors of lifestyle diseases (sedentariness, physical inactivity, poor cardiorespiratory fitness, suboptimal weight and body composition, metabolic syndrome markers). The analyses were also adjusted for participants’ sex, age, educational level and experiences of health, as these factors have also shown to associate with individuals’ preferences and health behavioral tendencies [37–41]. Due to the absence of previous studies on our topic, we based our hypotheses on the literature on the positive effects of music on health and well-being. In addition to these direct effects of music listening on health, music can encourage and enhance exercise, which could lead to better health outcomes. Thus, we hypothesised that use of music in mood-regulation would be positively associated with physical activity and cardiorespiratory fitness and negatively associated with cardiovascular risk factors.

Given the substantial public health implications and economic burden associated with overweight and physical inactivity, coupled with the detrimental effects on individual quality of life, investigating the potential of music as a possible preventive and/or intervention tool for these issues is important. While there is some evidence of possible maladaptive use of music as a tool for emotion regulation [42,43], the positive effects of music on health and exercise are widely described in the literature. We explored further the potential modification effects of sex, as the understanding of biologically determined variability between males and females might shed light on physical activity guidelines and sex-specific health care development [38,41].

This study was focused on examining these questions among overweight and obese adult men and women in Finland who participated in a lifestyle intervention initiative (Motivation Makes the Move—MoMaMo!) with the goal of reducing obesity, sedentary lifestyles, and the associated adverse health outcomes while exploring, among other solutions, the potential of music as a tool in lifestyle interventions. A better understanding of the relationship between music use and exercise could aid the development of future interventions to reduce obesity and physical inactivity.

## Methods

This study was part of a multi-disciplinary initiative called Motivation Makes the Move - MoMaMo!. Data collection for MoMaMo! took place in 2016–2020. The aim of the initiative was to reduce obesity, sedentary lifestyles, and associated adverse health outcomes in overweight and obese adults who were physically inactive. MoMaMo! aimed also to develop innovative and achievable means of individual lifestyle changes. The emotional use of music in everyday life (music as a means of mood and emotional regulation) and the possibilities of using music as a tool in a lifestyle intervention were explored as a part of this initiative.

MoMaMo! was registered at ClinicalTrials.gov (protocol record TYH2016215, NCT02686502). The study protocol was approved by the Ethics Committee (approval number 384/13/03/00/2015) of the Hospital District of Helsinki and Uusimaa, Helsinki, Finland. All experiments were performed in accordance with relevant guidelines and regulations. Written informed consent was obtained from all subjects after they received the research-related information.

### Participants

The study participants were recruited to MoMaMo! when the study was launched from different health care institutions in the Helsinki metropolitan area (such as public healthcare clinics, occupational healthcare, student health services). The recruitment was active between February 1, 2016 and September 30, 2019.

A call to participate in the study was targeted at overweight and obese (BMI ≥ 27.5 kg/m^2^) adults, both male and female, between 18 and 40 years of age. The cutoff for BMI was chosen based on the key paper from The Global BMI Mortality Collaboration [44] where hazard ratios for BMI ≥ 27.5 were greater than for BMI 25.0–27.5. In addition, setting the cut-off value to 27.5 was estimated to reduce recruitment of subjects in whom elevated BMI was due to extensive muscle mass. The participants were required to meet the inclusion criteria (BMI, referral from a physician to consult lifestyle clinic due to physical inactivity and overweight/obesity, non-smokers, deemed suitable for exercise testing and training by a physician). Exclusion criteria included the presence of a neurological or psychiatric disorder, use of medication influencing glucose homeostasis (except insulin) or autonomic nervous system function (e.g., β-blockers or selective serotonin reuptake inhibitors), pregnancy, physical disability, substance abuse, significant co-operation difficulties, and severe anaemia.

A total of 145 people were assessed for eligibility after expressing interest to join the initiative. Of these, 56 were either excluded for not meeting the inclusion criteria or did not report to the laboratory for the measurements. After this, 89 participants participated in the initial measurements. Of these, 76 participated in the cardiopulmonary exercise test. As we were interested in measured VO_2max_, only participants who participated in the cardiopulmonary exercise test were included in this study. In the initial power calculations for the MoMaMo! study, a sample size of 99 was determined to be sufficient to compare the different intervention groups, including the expected 30% attrition rate (see calculations in [45]).

The average age of the participants (n = 76) was 32.6 years old in the final sample for this study. There were 30 male and 46 female participants. Descriptive data of the subjects is presented in Table 1 in S1 Appendix: 32.9% (n = 73) of the participants had a university degree, 42.5% a university of applied sciences degree and 24.7% had an upper secondary or lower level degree. In the entire sample, the average BMI was 33.9 kg/m^2^, the average fat percentage was 39.8%, and the average visceral fat area was 158.8 cm^2^.

### Measurement methods

Due to the multi-disciplinary nature of the initiative, participants of MoMaMo! participated in a variety of measurements, tests, and assessments. Only the measurements vital to this study are presented in below. All measurements are described at MoMaMo! The study was registered at ClinicalTrials.gov (protocol record TYH2016215, NCT02686502).

#### Emotional use of music in everyday life.

The emotional use of music in everyday life was assessed with The Brief Music in Mood Regulation Scale (B-MMR) that is a shortened version of The Music in Mood Regulation Scale, one of the most widely used instruments to measure music-related mood-regulation strategies. The short version has shown reliability and the scores correlate well with measures of general emotional regulation and music-related activities [46]. Here, the participants answered 21 statements about their music use (e.g., “When I am distressed by something, music helps me to clarify my feelings”) on a 5-point Likert scale (1 = strongly disagree, 5 = strongly agree). A sum of the total 21 responses was calculated and then divided by 21 to obtain a mean score for the tendency of the participant to use music as a tool of mood-regulation. Mean variables were also calculated for the seven mood-regulation strategies (*entertainment, revival, strong sensation, diversion, discharge, mental work*, and *solace*). These mood-regulation strategies describe *how* music is used for emotional regulation; see Saarikallio [47] for a detailed description of these strategies.

#### Body composition.

An InBody720 device (Biospace Co., Ltd., Seoul, South Korea) was used to measure body composition by the bioimpedance method. The outcome variables chosen to represent body composition in this study were body mass index (BMI, kg/m^2^), body fat percentage (BFP, %), visceral fat area (VFA, cm^2^), and fat-free mass (FFM, kg).

#### Physical activity.

Participants completed a self-assessment questionnaire on physical activity (The International Physical Activity Questionnaire, IPAQ, long version). IPAQ has been shown to have acceptable validity when assessing physical activity in healthy adults [48]. The questionnaire considers physical activity in the following life domains: work, transport, housework/gardening, and leisure and differentiates between walking, cycling, moderate activity, and vigorous activity by giving them a different MET value. The total amount of a person’s physical activity (*MET minutes/week*) was calculated by multiplying the time spent on a certain activity by the corresponding MET value and adding these weighed activity time variables together [49].

#### Exercise performance, maximal oxygen uptake, and classification of cardiorespiratory fitness.

A physician examined each participant to ensure their suitability for exercise testing and training. Thereafter, each participant performed a step-incremental cardiopulmonary exercise test on a cycle ergometer (Monark Ergomedic 839 E, Monark Exercise AB) until voluntary fatigue. Data included recordings of pulmonary ventilation (Triple V, Jaeger Mijnhardt), alveolar gas exchange (Oxycon Pro), electrocardiography (ECG; PowerLab, AD Instruments), and the ratio of perceived exertion (RPE).

Exercise performance was determined based on the maximum workload achieved (*W, W/kg*). VO_2max_ was assessed with breath-by-breath alveolar gas exchange and pulmonary ventilation measurements. The highest 30-second moving average was calculated to obtain the absolute VO_2max_ (*l/min*). VO_2max_ was also calculated as a relative rate in millilitres of oxygen per kilogram of body mass per minute *(ml/kg/min*) and as millilitres of oxygen per kilogram of fat-free mass per minute (*ml/kg·FFM/min*). Scaling VO_2max_ to FFM takes body composition into account by excluding the fat mass, which is practically inactive during exercise. This is a common procedure to evaluate cardiorespiratory fitness especially in overweight and obese subjects [50,51]. These methods of exercise testing are routinely used in exercise laboratories [51–54].

VO_2max_ (*ml/kg/min*) measured during CPET can also be expressed as a cardiorespiratory fitness classification. Based on the norms of Shvartz and Reibold [55], each participant was categorized in a numbered class (1 to 7, where 1 = very poor and 7 = excellent). This classification considers the variation in VO_2max_ due to age and sex. In this study, most of the subjects were in poor condition due to the admission criteria, which meant that the distribution into different fitness categories was not even, and highest categories had no observations. There is evidence to suggest that even a small difference in VO_2max_ has an effect on cardiovascular risk factors [56]. Because of this and the practical reason of having very few or no observations in higher categories, in this study the classification was changed to “fitness class 1” and “fitness class 2 or higher”.

#### Other measurements.

A background information questionnaire was used to gather information about the participants’ education level, general health status, and possible medications. To assess the participants’ own experience of their general health status, the first question of RAND-36-item health survey was used to create a variable representing this. To standardise the direction of the scales across all variables, we reversed the scale of RAND-36-Q1: originally, this variable was coded on a 5-point Likert scale where 1 = “excellent” and 5 = “poor”. For consistency and interpretability, the scale was inverted so that higher scores correspond to more favourable outcomes.

Fasting blood samples were taken to analyse the inflammation marker hs-CRP (high-sensitivity C-reactive protein) and fasting glucose and insulin. Based on insulin and glucose values, the Homeostatic Model Assessment for Insulin Resistance (HOMA-IR) index was calculated [(serum insulin µ U/ml x fasting plasma glucose mmol/l) ÷ 22.5] [57]. Hs-CRP and HOMA-IR index were treated as risk factors of lifestyle diseases and metabolic syndrome biomarkers in this study. An actual classification of a participant having or not having metabolic syndrome was not performed.

### Statistical analyses

Descriptive statistics were calculated for key variables. Numeral and visual assessment revealed non-normality in the variables of self-assessed physical activity (IPAQ) and high-sensitivity C-reactive protein. For these two variables, a logarithmic transformation was applied to improve normality, allowing for the use of parametric analyses.

The connections between emotional use of music in everyday life and risk factors of lifestyle diseases were explored using linear regression models. When examining the assumptions of linear regression analysis, we found supportive evidence of these assumptions being met. The analyses were adjusted for the participants’ sex, age, highest level of education and experienced health to test the robustness of the results. Furthermore, if the variable sex was found to modify the associations, the analyses were separately conducted among males and females.

Logistic regression was used to assess the relationship between emotional use of music and the binary fitness class. The analyses were adjusted for participant’s highest level of education and experienced health, since fitness classification already takes into account the sex and age of the participant. All analyses were conducted using SPSS version 28 (IBM Corp).

## Results

### Emotional use of music

The averages and standard deviations for the B-MMR mean score and the seven mood-regulation strategies (all participants and men and women separately) are presented in Table 2 in S1 Appendix. The highest average was observed for the mood-regulation strategy *entertainment* (Table 2 in S1 Appendix).

### Physical activity, experienced health, cardiorespiratory fitness, and blood assays

Self-reported physical activity, experienced health, cardiorespiratory fitness, and results of the blood assays are presented separately for men and women in Table 3 in S1 Appendix.

Self-reported physical activity ranged from being completely physically inactive (IPAQ = 66 MET min/week) to highly active (IPAQ = 20 317 MET min/week). In a week, 20 317 MET minutes could mean for example about 55 hours of brisk walking [58]. The average woman in this study (4330.61 met min/week) would be classified as highly physically active and the average man (2541.17 met min/week) as moderately physically active [49]. Participants rated their health on a 1 (poor) to 5 (excellent) Likert scale to be 2.88 on average.

Based on cardiovascular data, in the whole sample, almost half (48.7%) of the participants were in the lowest fitness classification (class 1) and 28.9% were in the second lowest class (class 2). Only 14 participants (18.4%) were in fitness class 3, 2 participants were in fitness class 4, and 1 participant was in class 5.

Both for men and women, the average hs-CRP and HOMA-IR index were above the reference values (Hs-CRP: > 2.5 for men and > 3 for women [59]; HOMA-IR, > 2 for men and > 2.5 for women [60]).

### Connections between emotional use of music in everyday life and risk factors of lifestyle diseases

The results of linear regression analyses predicting body composition, physical activity, cardiorespiratory fitness, and blood assay results with emotional use of music in everyday life are shown in Table 1. Emotional use of music in everyday life was a statistically significant predictor of fat percentage (β=0.25, p = .027, R^2^ = 6.5%). Emotional use of music did not predict any of the other examined risk factors of lifestyle diseases.

**Table 1:** Emotional use of music predicting risk factors of lifestyle diseases.

Predicted variable	B	SE	β	t-value	p-value	R^2^	F-statistic (df)	p-value (F)
VO2max relative to FFM	−0.66	0.98	−0.08	−0.68	.501	0.6%	0.46 (1, 74)	.501
VO2 at VT1 relative to FFM	0.69	0.66	0.12	1.05	.298	1.5%	1.10 (1, 74)	.298
VO2 at VT2 relative to FFM^a^	0.50	0.79	0.07	0.63	.532	0.5%	0.40 (1, 73)	.532
body mass index	1.32	0.71	0.21	1.86	.067	4.5%	3.45 (1, 74)	.067
fat percentage	2.45	1.09	0.25	2.26	.027^*^	6.5%	5.11 (1, 74)	.027^*^
visceral fat area	9.79	6.24	0.18	1.57	.121	3.2%	2.46 (1, 74)	.121
high-sensitivity C-reactive protein (transformed)^b^	0.19	0.17	0.13	1.10	.275	1.7%	1.21 (1, 71)	.275
HOMA-IR index^c^	0.13	0.31	0.05	0.42	.674	0.2%	0.18 (1, 72)	.674
IPAQ self-reported physical activity (transformed)^a^	0.10	0.14	0.08	0.70	.484	0.7%	0.50 (1, 73)	.484

VO_2max_, maximal oxygen uptake; FFM, fat free mass; VT1/2, ventilatory threshold 1/2; HOMA-IR, Homeostatic Model Assessment for Insulin Resistance.

*p < .05 ^a^n = 75 ^b^n = 73 ^c^n = 74

When all the covariates (sex, age, education and experienced health) were adjusted for in the model, the association between emotional use of music and fat percentage attenuated (β=0.13, p = .227, Table 4 in S1 Appendix). When examining which covariate modified the association between emotional use of music and fat percentage, we found that sex (β=0.13, p = .058), age (β=0.23, p = .050) and educational level (β=0.19, p = .113) attenuated the association.

Based on this finding, we ran the analyses separately for men and women. In women, emotional use of music predicted fat percentage (β=0.38, p = .009, R^2^ = 14.5%) (Table 2). When adjusting for age, education and experienced health, music’s effect on fat percentage did not remain significant in women (β=0.22, p = .167) (Table 3). When examining which covariate modified the association between emotional use of music and fat percentage in women, we found that experienced health (β=0.25, p = .072) attenuated the association (Table 5 in S1 Appendix). Emotional use of music did not predict fat percentage in men (β=0.00, p = .981, R^2^ = 0.0%).

**Table 2:** B-MMR total score and mood-regulation strategies predicting fat percentage in women.

Predictor variable	B	SE	β	t-value	p-value	R^2^
B-MMR total score	2.68	0.98	0.38	2.73	.009**	14.5%
Entertainment	1.93	0.74	0.37	2.61	.012*	13.4%
Revival	1.74	0.79	0.32	2.20	.033*	9.9%
Strong sensation	1.81	0.86	0.30	2.10	.042*	9.1%
Diversion	2.12	0.82	0.37	2.60	.013*	13.3%
Discharge	0.92	0.73	0.19	1.27	.213	3.5%
Mental work	1.60	0.79	0.29	2.03	.049*	8.5%
Solace	2.01	0.92	0.31	2.19	.034*	9.8%

**p < .01.

*p < .05.

**Table 3:** B-MMR total score and mood-regulation strategies predicting fat percentage in women after adjusting for covariates.

Predictor variable	B	SE	β	t-value	p-value	R^2^
B-MMR total score	1.53	1.09	0.22	1.41	.167	24.9%
Entertainment	1.01	0.79	0.20	1.27	.212	24.2%
Revival	0.67	0.86	0.13	0.78	.440	22.2%
Strong sensation	0.87	0.92	0.14	0.94	.354	22.8%
Diversion	1.48	0.84	0.26	1.77	.084	27.0%
Mental work	0.55	0.89	0.10	0.62	.539	21.8%
Solace	1.09	0.99	0.17	1.10	.278	23.4%
entertainment adjusting for age: β=0.33, p = .029* entertainment adjusting for education: β=0.30, p = .050 entertainment adjusting for experienced health: β=0.24, p = .097 revival adjusting for age: β=0.27, p = .083 revival adjusting for education: β=0.23, p = .163 revival adjusting for experienced health: β=0.17, p = .241 strong sensation adjusting for age: β=0.26, p = .086 strong sensation adjusting for education: β=0.23, p = .129 strong sensation adjusting for experienced health: β=0.19, p = .188 diversion adjusting for age: β=0.33, p = .028* diversion adjusting for education: β=0.30, p = .045* diversion adjusting for experienced health: β=0.28, p = .039* mental work adjusting for age: β=0.25, p = .105 mental work adjusting for education: β=0.22, p = .161 mental work adjusting for experienced health: β=0.15, p = .306 solace adjusting for age: β=0.27, p = .076 solace adjusting for education: β=0.23, p = .128 solace adjusting for experienced health: β=0.22, p = 119						

*p < .05.

We also examined which of the seven mood regulation strategies were connected to fat percentage in women. These analyses are presented in Table 2. Every mood-regulation strategy except for discharge was a statistically significant predictor of fat percentage. When adjusting these six analyses for age, education and experienced health, these connections did not remain statistically significant (all p-values p > .05) (Table 3). When examining which covariate modified the association between each mood regulation strategy and fat percentage in women, we found that for *revival*, *strong sensation*, *mental work* and *solace*, age, educational level and experienced health all attenuated the association. For *entertainment*, only educational level and experienced health attenuated the association. For *diversion*, none of the covariates alone modified the association, but when adjusting for all the covariates, the association did not remain significant (p = .084). (Tables 6–11 in S1 Appendix.)

### Connections between emotional use of music in everyday life and fitness class

Emotional use of music in everyday life was associated with fitness class (B = −0.75, OR = 0.47, 95% CI = 0.26–0.88, p = .018) (Table 4). Addition of one point in emotional music use meant a 53% lower chance to be included in fitness class 2 or higher; thus higher scores in emotional music use were connected to a poorer fitness classification. When adjusting the analysis for education and experienced health, the connection between emotional use of music and fitness class remained robust (B = −0.68, OR = 0.51, 95% CI = 0.26–0.98, p = .044) (Table 5). Estimates for each confounder in this model can be found in Table 12 in S1 Appendix.

**Table 4:** B-MMR total score and mood-regulation strategies predicting fitness class.

Predictor variable	OR (95% CI)	P	Nagelkerke R^2^
B-MMR total score	0.47 (0.26–0.88)	.018*	10.6%
Entertainment	0.54 (0.32–0.90)	.018*	11.1%
Revival	0.61 (0.38–0.97)	.037*	8.0%
Strong sensation	0.63 (0.38–1.05)	.078	5.6%
Diversion	0.53 (0.32–0.86)	.011*	11.9%
Discharge	0.85 (0.58–1.26)	.423	1.1%
Mental work	0.68 (0.45–1.04)	.074	5.8%
Solace	0.64 (0.40–1.01)	.053	6.8%

*p < .05.

**Table 5:** B-MMR total score and mood-regulation strategies predicting fitness class after adjusting for covariates.

Predictor variable	OR (95% CI)	P	Nagelkerke R^2^
B-MMR total score	0.51 (0.26–0.98)	.044*	10.8%
Entertainment	0.56 (0.33–0.95)	.032*	12.3%
Revival	0.65 (0.40–1.07)	.089	8.4%
Diversion	0.55 (0.33–0.93)	.025*	12.3%

*p < .05.

Based on the significant result of the total B-MMR score predicting fitness class, the seven different mood-regulation strategies were also examined as a predictor of fitness class. These results are presented in Table 4. *Entertainment* (B = −0.62, OR = 0.54, 95% CI = 0.32–0.90, p = .018), *revival* (B = −0.50, OR = 0.61, 95% CI = 0.38–0.97, p = .037), and *diversion* (B = −0.64, OR = 0.53, 95% CI = 0.32–0.86, p = .011) were significant predictors.

The associations between *entertainment* and fitness class (B = −0.58, OR = 0.56, 95% CI = 0.33–0.95, p = .032) and *diversion* and fitness class (B = −0.59, OR = 0.55, 95% CI = 0.33–0.93, p = .025) remained robust after adjusting for education and experienced health. For *revival*, educational level (B = −0.42, OR = 0.66, 95% CI = 0.41–1.06, p = .084) and experienced health (B = −0.47, OR = 0.63, 95% CI = 0.39–1.01, p = .057) both attenuated the association. Estimates for each confounder in these models can be found in Tables 13–15 in S1 Appendix.

## Discussion

The aim of this study was to investigate the relationship between emotional use of music and the risk factors of lifestyle diseases, especially physical inactivity, poor cardiorespiratory fitness, overweight, and obesity. Our sample consisted of Finnish obese and overweight adults (aged 19 to 40, average BMI 33.9) who had signed up for a lifestyle intervention study and participated in the initial measurements.

Our results suggest that using music as a tool for emotional regulation is associated with poor cardiorespiratory fitness. This relationship remained significant after adjusting for participants’ educational level and their experienced general health. Emotional use of music was also positively associated with fat percentage, but sex, age and educational level attenuated the association. When examining the sexes separately, the association between emotional use of music and fat percentage was only found in women. When examining this further, we found that experienced health attenuated this association.

### Body composition, cardiorespiratory fitness, and physical activity

In our sample, the participants’ average fat percentage [61] and average visceral fat area [62] were both above the recommended values. The participants’ cardiorespiratory fitness was poor; almost half (48.7%) of the participants were in the lowest fitness classification (class 1) and 28.9% were in the second lowest class (class 2). Only a few participants in this study were classified in the average (class 4) category of higher.

Interestingly, despite being in poor shape and the study being aimed at people leading a sedentary lifestyle, self-reported physical activity was moderate or high. Self-reported physical activity ranged from completely physically inactive to people reporting around 8 hours of moderate physical activity per day, indicating considerable variability between participants. It seems plausible that the participants of this study overestimated the amount and intensity of physical activity in their everyday life. There is evidence of obese people overestimating their physical activity and especially the intensity of physical activity with the IPAQ [63]. In another study [64], when participants were allowed to expand on their IPAQ answers after an interview, their estimations about their physical activity were much lower.

### Emotional use of music in every day life and its association with risk factors of lifestyle diseases

Our results showed that emotional use of music in everyday life was negatively correlated with physical health. Although no statistically significant connections were found between music use and body mass index, visceral fat area, physical activity, and blood assays, we observed a positive relationship between emotional music use and body fat percentage. This relationship did not remain significant after controlling for participants’ age, educational level and their experienced general health. We also observed a negative association between emotional use of music and cardiorespiratory fitness; the higher the B-MMR score, the greater the risk of being classified as fitness class 1. This finding goes against our hypothesis of music being connected to favourable health outcomes through music’s positive influence on health and exercise. Our findings merely demonstrate a connection between poorer health and greater emotional use of music. Based on the current data we can not determine if such music-based emotion regulation is helpful for these individuals.

This surprising result might indicate people in poor physical condition being in greater need of emotional regulation tools, such as music. Poor cardiorespiratory fitness might also lead to higher experience of stress [65], and stress has been connected to emotional use of music [66]. A possible explanation for this result could also be that individuals engaging in music regularly do music instead of sports and exercise; they invest more time in the hobby of music than the hobby of exercising. We speculate that when deciding how to use their limited free time, some people might choose music over exercise and thus have less time for exercise and sports. In this study, we did not examine any other mood-regulation methods or hobbies than music, so we can not speculate on the possible effects of other sedentary activities. Another possibility could be that if the primary strategy of emotion regulation is movement and exercise, such individuals do not “need” music to regulate their emotions and vice versa. There is evidence of regular exercise being linked to better coping mechanisms and emotional resilience [67]. However, in our study, a connection was found between cardiorespiratory fitness and music use, and not between self-reported physical activity and music use.

Of the seven different mood-regulation strategies measured by B-MMR, e*ntertainment*, *revival*, and *diversion* were connected to fitness class. Of these relationships, associations between fitness class and *entertainment* and *diversion* remained significant after adjusting for confounders. These findings suggest that using music as a way to create a happy atmosphere (*entertainment*), to feel refreshed and get new energy (*revival*), and to turn one’s mind away from unpleasant thoughts or emotions (*diversion*) are linked to the lowest fitness class. The mood-regulation strategy *diversion* resembles the *distraction hypothesis* [68]; the idea that some of the mood-regulation and mental health benefits of exercise could be due to a “mental break” from worries and unpleasant emotions. The similarity between these two concepts could lend support to the idea that people use either music or exercise to distract themselves, which could then lead to the “music users” being in poorer shape than the “exercisers”.

A connection between body composition and music use was observed since the total B-MMR score was positively linked to fat percentage. This relationship did not remain significant after adjusting for confounders, and it was revealed that sex was a strong predictor of fat percentage. This finding is in line with previous research which suggests that women usually have a higher fat percentage than men [69]. We made the decision to examine the relationship between emotional music use and fat percentage separately in men and women to take this difference into account. The separate analyses revealed that B-MMR was also positively linked to fat percentage in women, but not in men. When adjusting for age, education and experienced health, music’s effect on fat percentage did not remain significant in women. When examining which covariate modified the association between emotional use of music and fat percentage in women, we found that experienced health attenuated the association.

A proposed mechanism of this relationship between emotional use of music and fat percentage could be the same as mentioned before, namely two different hobbies (music vs. exercise) competing for time or a difference in the preferred emotion regulation strategy (music vs. exercise). Body adiposity is also affected by diet and eating habits; it may also be possible that people experiencing negative emotions and using music to cope with them might also engage in emotional eating. In one study, the tendency to eat in response to negative emotions was connected to increased fat mass, and women also reported significantly higher levels of emotional eating than men [70]. There is also evidence from a study that emotional eating and using music as a mood-regulation tool, specifically the strategy of *discharge*, were connected [71].

One practical implementation of these results could be in designing future interventions for overweight, obese, and sedentary people. While emotional use of music was connected to unfavourable fitness outcomes in our sample, the causal relationship between these two remains to be investigated. It would be useful to consider the different ways that people regulate their mood and emotions when planning a lifestyle change, an exercise program, or both. For people who use a lot of music to regulate their mood, it may be beneficial to try to find ways to combine use of music with exercise or find alternative means of mood-regulation that involve physical activity. For individuals who have a tendency to seek consolation as their regulatory strategy, it might be beneficial to substitute possible emotional eating with musical solace. Increasing knowledge on the possibilities might make a difference, as increased self-awareness of one’s self-regulation and listening styles mediates the relationship between the intentions and outcomes of musical emotion regulation [72]. In our study, emotional use of music was linked to being in the lowest possible classification of cardiorespiratory fitness. Since even a small change in VO_2max_ can lower cardiovascular risk factors [56], finding ways to improve the cardiorespiratory fitness of the most unfit group is especially important.

### Limitations of this study

As discussed earlier, there were possible reliability issues with self-reported physical activity; it seems likely that physical activity was widely overestimated by the participants. One of the inclusion criteria of this study was physical inactivity, and it seems unlikely that participants would have increased their physical activity massively between the intake and the initial study measurements. It is possible that the results of the IPAQ would have been more reflective of the actual amount of physical activity of the participants if the questionnaire was completed in an interview with an exercise clinician or another expert. There is a wide range of answers and higher than expected averages of self-reported physical activity. Due to these possible challenges in the measuring process, the results from IPAQ should be interpreted with caution.

The participants of this study were a highly selected group; they were obese and overweight Finnish adults between the ages of 19 and 40 years, wanted to participate in a lifestyle intervention, and were able to take time out of their everyday life to visit the clinic multiple times. Most participants were women, and the connection between fat percentage and emotional use of music was only found in women when the sexes were examined separately. We must be cautious not to emphasize gender differences but rather acknowledge that our sample more reliably represents females than males. It is of note that women and men may differ in their self-reports regarding emotional processes. Our results may also be specific to obese and overweight individuals.

We recognise that the sample size of this study (n = 76) is relatively small, which affects the interpretation of the results. For future research, we suggest examining the relationship between emotional music use and cardiorespiratory fitness and body composition in larger samples, preferable using longitudinal designs to facilitate the exploring of causal effects. In our study, we found that sex, age, educational level and experienced health modified some of the relationships between emotional use of music and cardiorespiratory fitness and body composition. We suggest future research to explore these effects further.

### Strengths of this study

The major strength of this study is that cardiovascular fitness was measured objectively. As seen with physical activity in this study, self-reporting and other indirect measurement methods can present problems with reliability and validity. Measuring VO_2max_ using a direct method (CPET), with extensive additional physiological measurements, requires time, financial resources, trained staff, and a test subject who agrees to pedal a bike until exhaustion. From this perspective, the sample size of this study (n = 76) can be considered high.

## Conclusions

Using music as a mood regulator is associated with unfavourable fitness outcomes among overweight and obese adults who joined a lifestyle intervention. Future research could address the relationship between emotional music use and cardiorespiratory fitness and body composition in a population sample. The underlying mechanisms between music use and health should also be further examined.

## Supporting information

S1 Appendix Tables 1–15. (DOCX)
