# Supplementary material for: Using music as a mood regulator in everyday life is associated with unfavourable health and fitness outcomes in overweight adults
Source: PLoS One. 2025 Feb 27;20(2):e0317607. doi: 10.1371/journal.pone.0317607 (PMC11867320; doi:10.1371/journal.pone.0317607)
Supplement: S1 Appendix — (DOCX) [file pone.0317607.s001.docx]

**S1 Appendix:** **Tables 1–15**

| *Table 1. Means, standard deviations and frequencies of age, educational level and anthropometric measurements* | | | | | | | | | |  |  |  |
| --- | --- | --- | --- | --- | --- | --- | --- | --- | --- | --- | --- | --- |
|  |  |  |  |  |  |  |  |  |  |  |  |  |
|  |  |  |  |  |  |  |  |  |  |  |  |  |
|  | Men | | |  |  |  | Women | | |  |  |  |
|  |  |  |  |  |  |  |  |  |  |  |  |  |
| **Variable** | Mean | SD | min | max | n | % | Mean | SD | min | max | n | % |
|  |  |  |  |  |  |  |  |  |  |  |  |  |
| **sex** |  |  |  |  | 30 | 40 |  |  |  |  | 46 | 61 |
| **level of highest education** ^a^ |  |  |  |  |  |  |  |  |  |  |  |  |
| upper secondary level |  |  |  |  | 5 | 17 |  |  |  |  | 13 | 30 |
| university of applied sciences degree |  |  |  |  | 11 | 37 |  |  |  |  | 20 | 47 |
| university degree |  |  |  |  | 14 | 47 |  |  |  |  | 10 | 23 |
|  |  |  |  |  |  |  |  |  |  |  |  |  |
| age (years) | 32.7 | 5.09 | 22 | 40 |  |  | 32.6 | 5.83 | 19 | 40 |  |  |
| body mass index (kg/m^2^) | 33.5 | 4.68 | 26.8 | 45.5 |  |  | 34.1 | 5.43 | 27.7 | 53.3 |  |  |
| fat percentage (%) | 34.0 | 7.22 | 21.8 | 48.1 |  |  | 43.6 | 5.93 | 33.2 | 55.3 |  |  |
| visceral fat area (cm^2^) | 166.8 | 41.43 | 89.4 | 262.4 |  |  | 153.6 | 46.76 | 80.9 | 318.7 |  |  |

^a^ n=73 due to missing questionnaire data

| *Table 2. Means and standard deviations of B-MMR mean scores and mood-regulation strategies* | | | | |  |  |  |
| --- | --- | --- | --- | --- | --- | --- | --- |
|  |  |  |  |  |  |  |  |
|  |  |  |  |  |  |  |  |
| **Variable** | **Mean (all)** | **SD** | **Mean (men)** | **SD** | **Mean (women)** | **SD** |  |
|  |  |  |  |  |  |  |  |
| Entertainment | 4.0 | 1.05 | 4.0 | 0.94 | 4.0 | 1.12 |  |
| Revival | 3.2 | 1.05 | 3.2 | 1.03 | 3.2 | 1.07 |  |
| Strong sensation | 3.7 | 0.94 | 3.6 | 0.87 | 3.7 | 0.99 |  |
| Diversion | 2.6 | 1.01 | 2.5 | 0.99 | 2.7 | 1.02 |  |
| Discharge | 2.3 | 1.18 | 2.1 | 1.14 | 2.4 | 1.21 |  |
| Mental work | 2.9 | 1.14 | 2.8 | 1.22 | 3.1 | 1.08 |  |
| Solace | 2.9 | 1.06 | 2.5 | 1.16 | 3.1 | 0.92 |  |
| B-MMR mean score | 3.1 | 0.82 | 3.0 | 0.79 | 3.2 | 0.84 |  |
| n | 76 |  | 30 |  | 46 |  |  |
| B-MMR = The Brief Music in Mood Regulation Scale |  |  |  |  |  |  |  |

| *Table 3. Means, standard deviations, frequencies, and group comparisons of physical activity, cardiorespiratory fitness, and blood assays* | | | | | | | | | |  |  |  |  |
| --- | --- | --- | --- | --- | --- | --- | --- | --- | --- | --- | --- | --- | --- |
|  |  |  |  |  |  |  |  |  |  |  |  |  |  |
|  |  |  |  |  |  |  |  |  |  |  |  |  |  |
|  | Men | | |  |  |  | Women | | |  |  |  |  |
|  |  |  |  |  |  |  |  |  |  |  |  |  |  |
| **Variable** | Mean | SD | min | max | n | % | Mean | SD | min | max | n | % |  |
|  |  |  |  |  |  |  |  |  |  |  |  |  |  |
| **fitness class** |  |  |  |  |  |  |  |  |  |  |  |  |  |
| class 1 |  |  |  |  | 14 | 47 |  |  |  |  | 23 | 50 |  |
| class 2 or higher |  |  |  |  | 16 | 53 |  |  |  |  | 23 | 50 |  |
|  |  |  |  |  |  |  |  |  |  |  |  |  |  |
| MET-min/week (IPAQ) ^a^ | 2541.2 | 2365.3 | 66 | 11538 |  |  | 4330.6 | 4748.9 | 180 | 20317 |  |  |  |
| VO_2max_ (l/min) | 3.20 | 0.58 | 2.34 | 4.29 |  |  | 2.29 | 0.42 | 1.38 | 3.32 |  |  |  |
| VO_2max_ (ml/kg/min) | 29.8 | 5.84 | 19.4 | 43.1 |  |  | 24.9 | 5.56 | 9.9 | 37.3 |  |  |  |
| VO_2max_ (ml/kg·FFM/min) | 45.0 | 6.63 | 32.3 | 61.3 |  |  | 43.8 | 7.22 | 21.7 | 60.2 |  |  |  |
| VT1 VO_2_ (ml/kg·FFM/min) | 24.0 | 4.66 | 15.0 | 34.8 |  |  | 24.3 | 4.75 | 16.0 | 37.6 |  |  |  |
| VT2 VO_2_ (ml/kg·FFM/min)^a^ | 36.7 | 4.98 | 27.1 | 48.7 |  |  | 36.6 | 5.93 | 21.2 | 50.1 |  |  |  |
| Hs-CRP ^b^ | 3.1 | 2.71 | 0.2 | 10.0 |  |  | 6.3 | 8.24 | 0.2 | 45.0 |  |  |  |
| HOMA-IR index ^c^ | 3.1 | 1.68 | 0.7 | 8.0 |  |  | 3.7 | 2.39 | 0.2 | 12.0 |  |  |  |
| Experienced health (RAND-36 Q1 reversed) | 2.8 | 0.77 | 2 | 5 |  |  | 3.0 | 0.82 | 1 | 4 |  |  |  |

^a^ n=75 due to missing questionnaire data or laboratory error ^b^ n=73 due to laboratory error ^c^ n=74 due to laboratory error

MET = metabolic equivalent of task; VO_2max_ = maximal oxygen uptake; FFM = fat free mass; VT1/2= ventilatory threshold 1/2; Hs-CRP = high-sensitivity C-reactive protein; HOMA-IR = Homeostatic Model Assessment for Insulin Resistance

IPAQ log transformation: Mean = 7.74, SD = 0.98, min = 4.19, max = 9.92

Hs-CRP log transformation: Mean = 0.99, SD = 1.16, min= -1.90, max = 3.81

| *Table 4. B-MMR total score predicting fat percentage after adjusting for covariates* | | | | | | |  |
| --- | --- | --- | --- | --- | --- | --- | --- |
|  | **B** | **SE** | **β** | **t-value** | **p-value** |  |  |
| Sex | 9.16 | 1.62 | 0.56 | 5.65 | .000 |  |  |
| Age | -0.04 | 0.16 | -0.03 | -0.26 | .798 |  |  |
| Education | -1.03 | 1.18 | -0.10 | -0.87 | .385 |  |  |
| Experienced health | 0.02 | 1.09 | 0.00 | 0.02 | .988 |  |  |
| B-MMR total score | 1.26 | 1.04 | 0.13 | 1.22 | .227 |  |  |

B-MMR total score adjusting for sex: β=0.13, p=.058

B-MMR total score adjusting for age: β=0.23, p=.050

B-MMR total score adjusting for education: β=0.19, p=.113

B-MMR total score adjusting for experienced health: β=0.27, p=.024

| *Table 5. B-MMR total score predicting fat percentage after adjusting for covariates in women* | | | | | | | | |
| --- | --- | --- | --- | --- | --- | --- | --- | --- |
|  | **B** | **SE** | **β** | **t-value** | **p-value** |  |  |  |
| Age | -0.05 | 0.17 | -0.05 | -0.27 | .790 |  |  |  |
| Education | -0.44 | 1.45 | -0.06 | -0.31 | .761 |  |  |  |
| Experienced health | -2.49 | 1.19 | -0.34 | -2.09 | .043 |  |  |  |
| B-MMR total score | 1.53 | 1.09 | 0.22 | 1.41 | .167 |  |  |  |

Model with B-MMR only: B=2.68, SE=0.98, β=0.38, t=2.73, p=.009, R²=14.5 %, F=7.46 (1, 44), p=.009 B-MMR total score adjusting for age: β=0.34, p=.027

B-MMR total score adjusting for education: β=0.31, p=.046

B-MMR total score adjusting for experienced health: β=0.25, p=.072

| *Table 6: Entertainment predicting fat percentage after adjusting for covariates in women* | | | | | | | |  |
| --- | --- | --- | --- | --- | --- | --- | --- | --- |
|  | **B** | **SE** | **β** | **t-value** | **p-value** |  |  |  |
| Age | -0.07 | 0.17 | -0.07 | -0.43 | .667 |  |  |  |
| Education | -0.40 | 1.45 | -0.05 | -0.27 | .786 |  |  |  |
| Experienced health | -2.48 | 1.21 | -0.34 | -2.04 | .048 |  |  |  |
| Entertainment | 1.01 | 0.79 | 0.20 | 1.27 | .212 |  |  |  |

entertainment adjusting for age: β=0.33, p=.029

entertainment adjusting for education: β=0.30, p=.050

entertainment adjusting for experienced health: β=0.24, p=.097

| *Table 7: Revival predicting fat percentage after adjusting for covariates in women* | | | | | | | |  |
| --- | --- | --- | --- | --- | --- | --- | --- | --- |
|  | **B** | **SE** | **β** | **t-value** | **p-value** |  |  |  |
| Age | -0.09 | 0.17 | -0.09 | -0.54 | .595 |  |  |  |
| Education | -0.26 | 1.48 | -0.03 | -0.17 | .863 |  |  |  |
| Experienced health | -2.73 | 1.21 | -0.37 | -2.26 | .030 |  |  |  |
| Revival | 0.67 | 0.86 | 0.13 | 0.78 | .440 |  |  |  |

revival adjusting for age: β=0.27, p=.083

revival adjusting for education: β=0.23, p=.163

revival adjusting for experienced health: β=0.17, p=.241

| *Table 8: Strong sensation predicting fat percentage after adjusting for covariates in women* | | | | | | | | |
| --- | --- | --- | --- | --- | --- | --- | --- | --- |
|  | **B** | **SE** | **β** | **t-value** | **p-value** |  |  |  |
| Age | -0.08 | 0.17 | -0.08 | -0.45 | .653 |  |  |  |
| Education | -0.57 | 1.48 | -0.07 | -0.38 | .704 |  |  |  |
| Experienced health | -2.69 | 1.20 | -0.36 | -2.24 | .031 |  |  |  |
| Strong sensation | 0.87 | 0.92 | 0.14 | 0.94 | .354 |  |  |  |

strong sensation adjusting for age: β=0.26, p=.086

strong sensation adjusting for education: β=0.23, p=.129

strong sensation adjusting for experienced health: β=0.19, p=.188

| *Table 9: Diversion predicting fat percentage after adjusting for covariates in women* | | | | | | | |  |
| --- | --- | --- | --- | --- | --- | --- | --- | --- |
|  | **B** | **SE** | **β** | **t-value** | **p-value** |  |  |  |
| Age | -0.07 | 0.17 | -0.07 | -0.39 | .697 |  |  |  |
| Education | -0.38 | 1.42 | -0.05 | -0.27 | .789 |  |  |  |
| Experienced health | -2.69 | 1.14 | -0.36 | -2.37 | .023 |  |  |  |
| Diversion | 1.48 | 0.84 | 0.26 | 1.77 | .084 |  |  |  |

diversion adjusting for age: β=0.33, p=.028

diversion adjusting for education: β=0.30, p=.045

diversion adjusting for experienced health: β=0.28, p=.039

| *Table 10: Mental work predicting fat percentage after adjusting for covariates in women* | | | | | | | |  |
| --- | --- | --- | --- | --- | --- | --- | --- | --- |
|  | **B** | **SE** | **β** | **t-value** | **p-value** |  |  |  |
| Age | -0.09 | 0.18 | -0.09 | -0.54 | .595 |  |  |  |
| Education | -0.41 | 1.48 | -0.05 | -0.28 | .784 |  |  |  |
| Experienced health | -2.72 | 1.24 | -0.37 | -2.20 | .034 |  |  |  |
| Mental work | 0.55 | 0.89 | 0.10 | 0.62 | .539 |  |  |  |

mental work adjusting for age: β=0.25, p=.105

mental work adjusting for education: β=0.22, p=.161

mental work adjusting for experienced health: β=0.15, p=.306

| *Table 11: Solace predicting fat percentage after adjusting for covariates in women* | | | | | | | |  |
| --- | --- | --- | --- | --- | --- | --- | --- | --- |
|  | **B** | **SE** | **β** | **t-value** | **p-value** |  |  |  |
| Age | -0.07 | 0.17 | -0.07 | -0.41 | .682 |  |  |  |
| Education | -0.51 | 1.46 | -0.06 | -0.35 | .732 |  |  |  |
| Experienced health | -2.73 | 1.18 | -0.37 | -2.32 | .026 |  |  |  |
| Solace | 1.09 | 0.99 | 0.17 | 1.10 | .278 |  |  |  |

solace adjusting for age: β=0.27, p=.076

solace adjusting for education: β=0.23, p=.128

solace adjusting for experienced health: β=0.22, p=119

| *Table 12: B-MMR total score predicting binary fitness class after adjusting for covariates* | | | | | |
| --- | --- | --- | --- | --- | --- |
|  | **OR** | **95 % CI lower upper** | | **p-value** |  |
| Education | 1.26 | 0.64 | 2.45 | .503 |  |
| Experienced health | 0.93 | 0.48 | 1.80 | .833 |  |
| B-MMR total score | 0.51 | 0.26 | 0.98 | .044 |  |
|  |  |  |  |  |  |

| *Table 13: Entertainment predicting binary fitness class after adjusting for covariates* | | | | | |
| --- | --- | --- | --- | --- | --- |
|  | **OR** | **95 % CI lower upper** | | **p-value** |  |
| Education | 1.26 | 0.64 | 2.47 | .505 |  |
| Experienced health | 1.06 | 0.55 | 2.03 | .864 |  |
| Entertainment | 0.56 | 0.33 | 0.95 | .032 |  |

| *Table 14: Revival predicting binary fitness class after adjusting for covariates* | | | | | |
| --- | --- | --- | --- | --- | --- |
|  | **OR** | **95 % CI lower upper** | | **p-value** |  |
| Education | 1.33 | 0.69 | 2.56 | .393 |  |
| Experienced health | 0.98 | 0.51 | 1.87 | .944 |  |
| Revival | 0.65 | 0.40 | 1.07 | .089 |  |

revival adjusting for education B=-0.42, OR=0.66, 95% CI=0.41–1.06, p=.084)
revival adjusting for experienced health B=-0.47, OR=0.63, 95% CI=0.39–1.01, p=.057

| *Table 15: Diversion predicting binary fitness class after adjusting for covariates* | | | | | |
| --- | --- | --- | --- | --- | --- |
|  | **OR** | **95 % CI lower upper** | | **p-value** |  |
| Education | 1.31 | 0.68 | 2.53 | .421 |  |
| Experienced health | 0.95 | 0.50 | 1.81 | .885 |  |
| Diversion | 0.55 | 0.33 | 0.93 | .025 |  |
